# Leniolisib reduced lymphoproliferative disease in murine autoimmune lymphoproliferative syndrome

**DOI:** 10.70962/jhi.20250125

**Published:** 2025-11-17

**Authors:** Chopie Hassan, Yolanda Ponstein, Robert Hanssen, Kevin S. Thorneloe, Rebecca A. Marsh, V. Koneti Rao

**Affiliations:** 1 Pharming Technologies BV, Leiden, Netherlands; 2 Pharming Healthcare, Inc, Warren, NJ, USA; 3 National Institute of Allergy and Infectious Diseases, National Institutes of Health, Bethesda, MD, USA

## Abstract

Autoimmune lymphoproliferative syndrome (ALPS) is an inborn error of immunity (IEI) characterized by abnormal FAS-mediated apoptosis of lymphocytes that leads to lymphoproliferation and expansion of CD4^−^/CD8^−^ double-negative T cells (DNTs). In patients with ALPS-FAS, DNTs have been reported to exhibit increased activity in the phosphoinositide 3-kinase δ (PI3Kδ)/mammalian target of rapamycin (mTOR) pathway. Although mTOR inhibition with sirolimus has improved autoimmune cytopenias and organomegaly in patients with ALPS, it requires monitoring of serum levels, and common adverse events frequently hamper long-term use. As leniolisib, a selective PI3Kδ inhibitor, reduced lymphoproliferation in another IEI known as activated PI3Kδ syndrome, efficacy was examined in a murine model of ALPS. Changes in organ weight and key immune subsets in MRL/lpr^−/−^ mice receiving vehicle or leniolisib (40 or 80 mg/kg/day) by oral gavage were assessed. Leniolisib limited the canonical features of ALPS, including lymphadenopathy, splenomegaly, and elevated DNTs, in a dose-dependent manner. These results support the evaluation of leniolisib in patients with ALPS (NCT06549114).

## Teaser

Treating patients with ALPS can be challenging. This article examines the efficacy of leniolisib, a selective PI3Kδ inhibitor, in a murine model of ALPS, illustrating a reduction in lymphoproliferation and disease-associated biomarkers such as elevated double-negative T cells.

## Introduction

Autoimmune lymphoproliferative syndrome (ALPS) is an inborn error of immunity (IEI) commonly caused by variants in *FAS*, the gene encoding the cell surface death receptor Fas. Germline or somatic variants in *FAS* can lead to apoptotic defects in lymphocytes, resulting in a subtype of ALPS known as ALPS-FAS (1, 2, 3). While germline variants in *FAS* are the most common, additional subtypes of ALPS have been reported in patients that are linked to other genetic variants such as *TNFSF6* and *CASP10*. Regardless of the subtype, this disorder usually presents in childhood with splenomegaly, nonmalignant lymphadenopathy, chronic cytopenias, and elevated CD4^−^/CD8^−^ double-negative T cells (DNTs) (4). Patients with ALPS-FAS also have an increased risk of developing B cell lymphomas (3).

In individuals with ALPS-FAS, DNTs have been reported to exhibit increased activity in the phosphoinositide 3-kinase (PI3K)/mammalian target of rapamycin (mTOR) pathway (5). Specifically, ex vivo studies demonstrated enhanced phosphorylation of Akt, mTOR, and S6 in DNTs from patients with ALPS compared with those from healthy controls. ALPS DNTs treated with the mTOR inhibitor sirolimus had reduced proliferation and increased apoptosis. As PI3Kδ is predominately expressed in lymphocytes, these results further support a role for PI3Kδ/mTOR in the development of aberrant DNTs. Collectively, the evidence supports that hyperactive PI3Kδ/mTOR signaling along with altered programmed cell death contributes to lymphoproliferation and expansion of CD4^−^/CD8^−^ DNTs in patients with ALPS-FAS (3, 5).

Treating ALPS can be challenging, especially in patients with refractory cytopenias and splenomegaly, the latter of which often develops at a young age. Currently, most therapeutics are either symptomatic or can produce serious or potentially severe adverse events (4). Sirolimus reduced organomegaly and improved autoimmune cytopenias in patients with ALPS; however, this treatment requires monitoring of serum levels and may lead to the development of oral mucositis and hyperlipidemia, which can result in discontinuation of treatment (6). It is cumbersome for clinicians to monitor and maintain trough levels of sirolimus in various patient populations, particularly in children who cannot swallow pills and require liquid formulations. Thus, there is a need for a treatment option that does not require strict monitoring due to a narrow therapeutic index, is well tolerated, and targets the multifaceted clinical components of ALPS. PI3Kδ is a potential therapeutic target that can be selectively inhibited with leniolisib, which is approved by the US Food and Drug Administration for patients ≥12 years of age with an IEI known as activated PI3Kδ syndrome (APDS) (7, 8).

Here, we assessed leniolisib treatment in MRL/lpr^−/−^ mice with pathogenic variants in the *lpr* gene located on chromosome 19, which is equivalent to human *FAS* on chromosome 10 (9, 10, 11). With virtually no detectable levels of fas protein, these mice develop an ALPS-like phenotype that includes autoimmunity, lymphadenopathy, splenomegaly, and expansion of CD4^−^/CD8^−^ DNTs and have been used historically to evaluate the potential benefit of other therapeutic candidates (11, 12, 13, 14, 15, 16, 17, 18, 19, 20).

## Results

**Figure 1.**
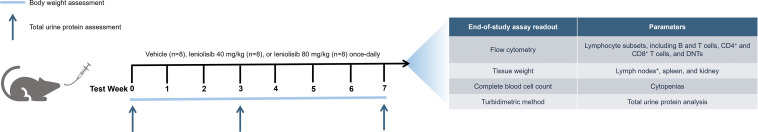
**Experimental timeline and parameters assessed during treatment with leniolisib or vehicle.** *Six lymph nodes were collected per mouse: two inguinal, two axillary, and two brachial.

The impact of leniolisib was assessed in a murine model of ALPS. Briefly, 6-wk-old female MRL/lpr^−/−^ mice received a daily dose of leniolisib (40 or 80 mg/kg) or vehicle (all groups, *n* = 8) by oral gavage for 7 wk (Fig. 1).

### Clinical observations

**Figure 2.**
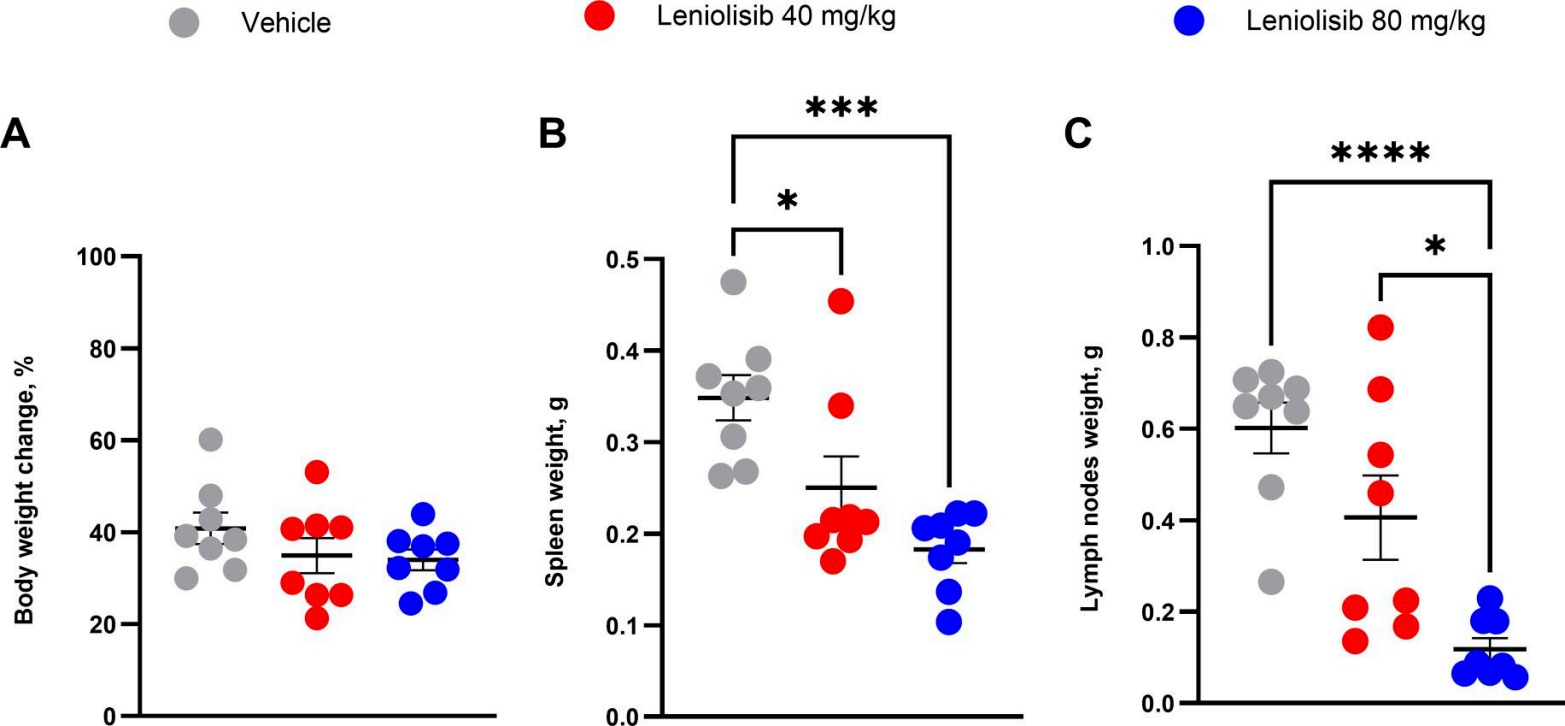
**Body weight change and organ weight at the end of the study. (A–C)** Individual and mean percent change in body weight from start weight (A). Individual and mean weight of spleen (B) and lymph nodes (C) following necropsy. Six lymph nodes (i.e., two inguinal, two axillary, and two brachial) from each mouse were used to determine weight (C). *n* = 8 per experimental group. Error bars are ± SEM. Data were analyzed using a one-way ANOVA with Tukey correction. *P < 0.05; ***P < 0.001; ****P < 0.0001.

No toxicities were observed in mice treated with leniolisib. Over the course of the study, mice did not develop any apparent infection or other clinical manifestations associated with immunosuppression. No statistically significant changes were noted in the percentage of live cells in the spleen, lymph nodes, blood, or bone marrow across all experimental groups. An increased number of palpable lymph nodes were observed in mice receiving vehicle compared with those receiving leniolisib. Nearly 17% of mice (three that received vehicle and one that received 80 mg/kg leniolisib) had skin lesions by the end of the study, supporting previous reports in the literature for this mouse model (21, 22). No significant differences between experimental groups in body weight changes from baseline weight were noted (Fig. 2 A). All dosing regimens were tolerated for the study duration.

### Leniolisib limited lymphoproliferation

Compared with vehicle, mice treated with leniolisib had a significantly lower spleen weight. Specifically, the average spleen weight of mice administered 40 mg/kg leniolisib was 0.250 g vs. 0.348 g (P = 0.0355) in the vehicle group and 0.183 g for mice administered 80 mg/kg leniolisib (P = 0.0005) (Fig. 2 B). This difference was also observed when spleen weight was normalized to body weight, as mean spleen weight of mice administered 80 mg/kg leniolisib was 0.548% of their body weight vs. 0.875% in mice treated with vehicle (P = 0.0469) (data not shown). In the 80 mg/kg leniolisib group, the weight of lymph nodes was 0.118 g vs. 0.602 g in the vehicle group (P < 0.0001). When normalized to body weight, the weight of lymph nodes in the 80 mg/kg leniolisib group was also significantly lower than the vehicle group (0.348% of body weight vs. 1.549%; P = 0.0027) (data not shown). Although not significant, the weight of lymph nodes was lower in mice treated with 40 mg/kg leniolisib compared with those administered vehicle (0.406 g vs. 0.602 g; P = 0.0983) (Fig. 2 C), which was also the case when normalized to body weight (1.234% of body weight vs. 1.549%; P = 0.5835) (data not shown). Overall, leniolisib limited both lymphadenopathy and splenomegaly in murine ALPS.

### Leniolisib limited expansion of aberrant immune subsets

At the end of the study, we assessed the percentage of live cells, absolute counts, and frequency (as a percentage of CD3^+^ or CD45^+^ cells) of CD4^−^/CD8^−^ DNTs and other immune subsets in the spleen, lymph nodes, blood, and bone marrow.

**Figure S1.**
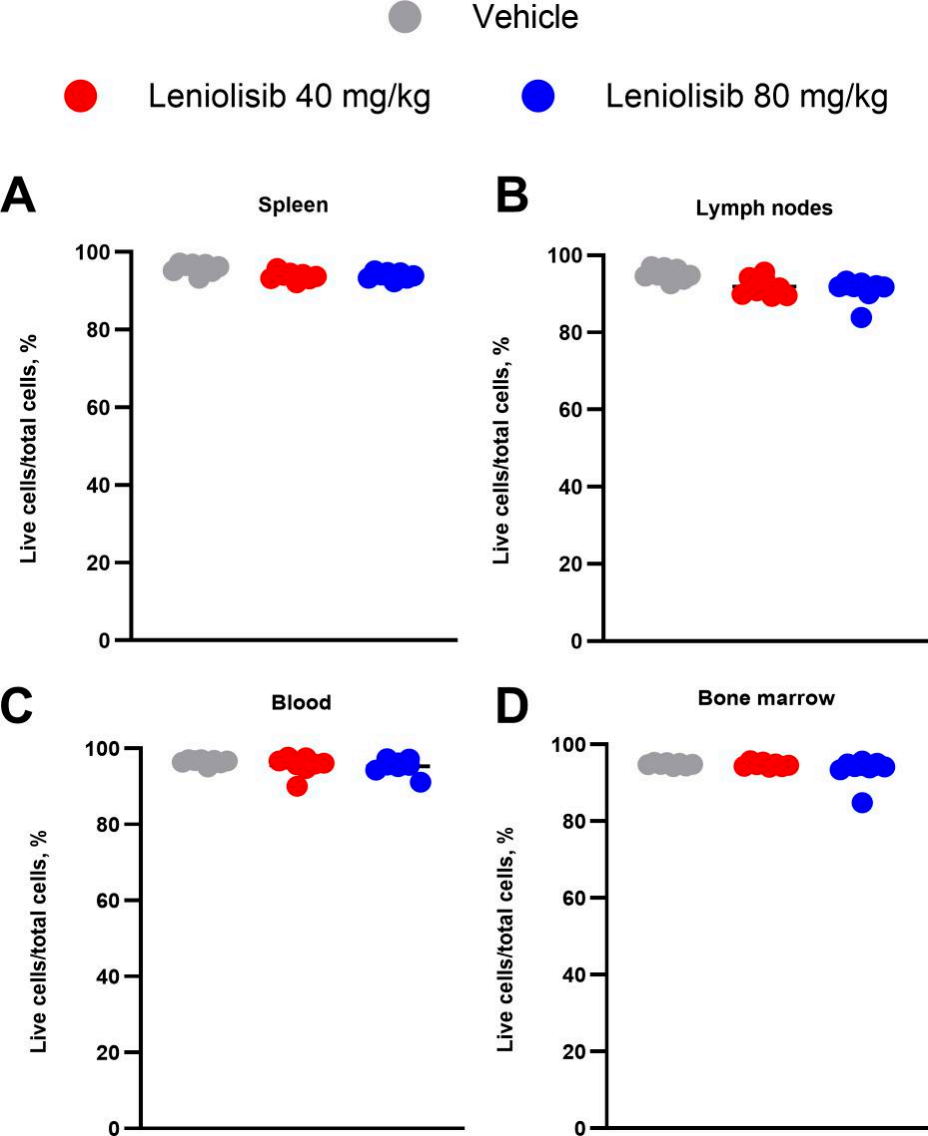
**Frequency of live cells at the end of the study. (A–D)** Frequency of live cells in the spleen (A), lymph nodes (B), blood (C), and bone marrow (D). Half of the spleen and three lymph nodes from the right side of each mouse were used for detection of live cells in A and B, respectively. *n* = 8 per experimental group. Error bars are ± SEM. Data were analyzed using a one-way ANOVA with Tukey correction (P < 0.05).

Overall, there was no change in the percentage of live cells out of total cells in the spleen, lymph nodes, blood, or bone marrow between all experimental groups (Fig. S1).

#### Spleen

**Figure 3.**
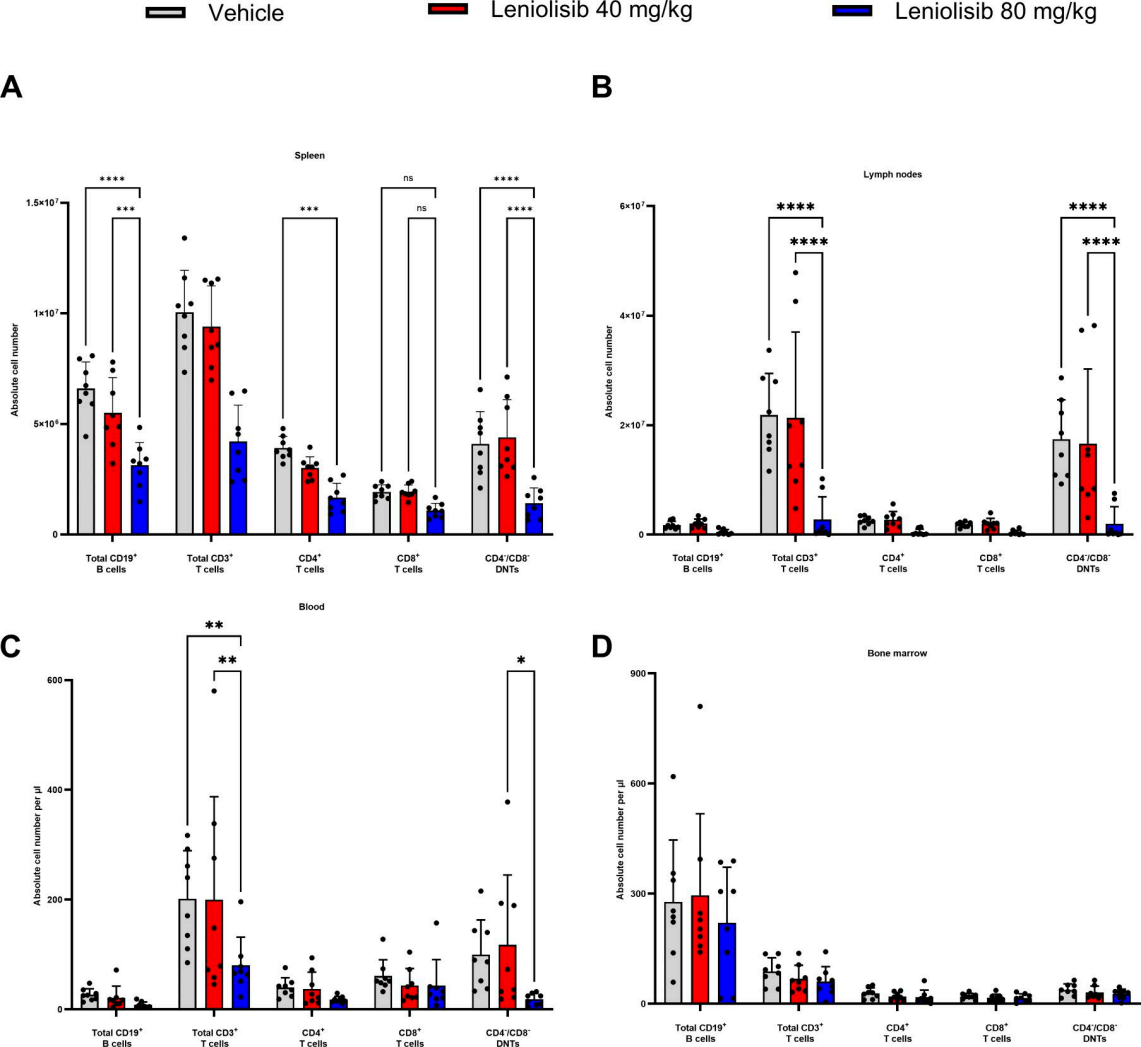
**Changes in immune subsets at the end of the study. (A–D)** Individual and mean absolute B and T cell counts in the spleen (A), lymph nodes (B), blood (C), and bone marrow (D). Half of the spleen (A) and three right-hand side lymph nodes (B) were processed for flow cytometry. *n* = 8 per experimental group. Error bars are ± SEM. Data were analyzed using a two-way ANOVA with Tukey correction. *P < 0.05; **P < 0.01; ***P < 0.001; ****P < 0.0001.

In the spleen (Fig. 3 A), absolute counts of CD4^−^/CD8^−^ DNTs were significantly lower in mice receiving 80 mg/kg leniolisib compared with mice administered vehicle (1.42 × 10^6^ cells vs. 4.10 × 10^6^ cells; P < 0.0001). Total CD3^+^ T cells were significantly lower in the 80 mg/kg leniolisib group compared with mice treated with vehicle (4.21 × 10^6^ cells vs. 10.06 × 10^6^ cells; P < 0.0001). Similarly, mice treated with 80 mg/kg leniolisib had significantly lower CD4^+^ T cells compared with those receiving vehicle (1.67 × 10^6^ cells vs. 3.92 × 10^6^ cells; P = 0.0008). In the B cell compartment, the 80 mg/kg leniolisib group had significantly lower absolute counts of CD19^+^ B cells compared with mice administered vehicle (3.15 × 10^6^ cells vs. 6.61 × 10^6^; P < 0.0001).

**Figure S2.**
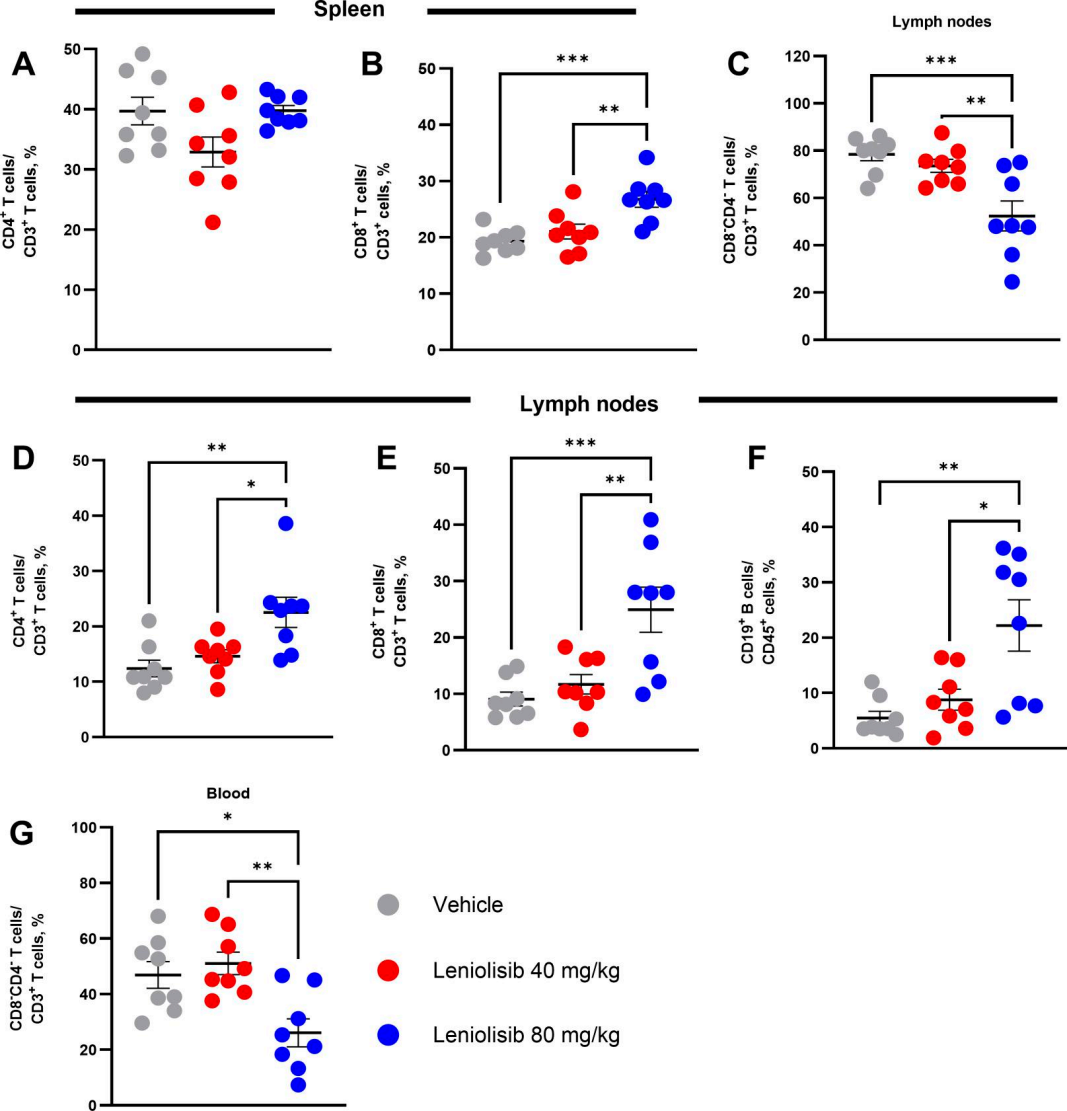
**Changes in frequency of select immune subsets at the end of the study. (A–G)** Individual and mean CD4^+^ (A) and CD8^+^ (B) T cell frequency in the spleen. Individual and mean CD8^−^/CD4^−^ DNT (C), CD4^+^ T cell (D), CD8^+^ T cell (E), and CD19^+^ B cell (F) frequencies in the lymph nodes. Individual and mean CD8^+^ T cell frequency in the blood (G). *n* = 8 per experimental group. Error bars are ± SEM. Data were analyzed using a one-way ANOVA with Tukey correction. *P < 0.05; **P < 0.01; ***P < 0.001.

Unlike absolute counts of CD4^+^ T cells, there was no change in the percentage of CD4^+^ T cells out of CD3^+^ cells in the spleen (Fig. S2 A) between experimental groups. Although there was no change in absolute counts of CD8^+^ T cells between experimental groups, the percentage of CD8^+^ T cells out of CD3^+^ cells in the spleen was significantly higher in mice treated with 80 mg/kg leniolisib (26.79% vs. 19.33%; P = 0.0007) (Fig. S2 B). Finally, no significant changes were observed in the frequency of CD19^+^ B cells, CD3^+^ T cells, or CD4^−^/CD8^−^ DNTs between spleens of experimental groups (data not shown).

#### Lymph nodes

In pooled analysis of lymph node samples (Fig. 3 B), which consisted of three lymph nodes from the right-hand side of each mouse (i.e., inguinal, brachial, and axillary), mice treated with 80 mg/kg leniolisib had significantly lower absolute counts of CD4^−^/CD8^−^ DNTs compared with mice administered vehicle (1.96 × 10^6^ cells vs. 17.43 × 10^6^ cells; P < 0.0001). Total CD3^+^ T cells were also significantly lower in the 80 mg/kg leniolisib group (2.75 × 10^6^ cells vs. 21.84 × 10^6^ cells; P < 0.0001). No statistically significant changes in absolute counts of B cells, CD4^+^ T cells, or CD8^+^ T cells in lymph nodes were noted between experimental groups.

Similar to absolute counts in pooled lymph node samples, mice treated with 80 mg/kg leniolisib had a significantly lower percentage of CD4^−^/CD8^−^ DNTs out of CD3^+^ T cells compared with mice receiving vehicle (52.39% vs. 78.48%; P = 0.0009) (Fig. S2 C). However, the percentages of CD4^+^ T cells, CD8^+^ T cells, and CD19^+^ B cells were significantly higher in mice administered leniolisib compared with mice administered vehicle. Specifically, the percentage of CD4^+^ T cells out of CD3^+^ T cells was significantly higher in mice administered 80 mg/kg leniolisib compared with those administered vehicle (22.53% vs. 12.39%; P = 0.0034) (Fig. S2 D). In the 80 mg/kg leniolisib group, the percentage of CD8^+^ T cells was higher compared with the vehicle group (24.94% vs. 9.07%; P = 0.0009) (Fig. S2 E). In the B cell compartment, mice treated with 80 mg/kg leniolisib had a significantly higher percentage of CD19^+^ B cells out of CD45^+^ cells compared with mice treated with vehicle (22.21% vs. 5.48%; P = 0.0019) (Fig. S2 F). No significant change was detected in the percentage of CD3^+^ T cells between experimental groups.

#### Blood

In terminal blood samples (Fig. 3 C), mice administered 80 mg/kg leniolisib had significantly lower absolute counts of total CD3^+^ T cells compared with mice receiving vehicle (80.32 cells/μl vs. 201.4 cells/μl; P = 0.0020). Although not significant when compared with mice administered vehicle, absolute CD4^−^/CD8^−^ DNTs were numerically lower in mice treated with 80 mg/kg leniolisib (18.47 cells/μl vs. 99.81 cells/μl; P = 0.0534). No significant changes were noted in absolute counts of B cells, CD4^+^ T cells, or CD8^+^ T cells between experimental groups.

Although not significant for absolute counts, the percentage of CD4^−^/CD8^−^ DNTs out of CD3^+^ T cells was significantly lower in blood of mice treated with 80 mg/kg leniolisib compared with mice receiving vehicle (26.09% vs. 46.91%; P = 0.0119) (Fig. S2 G). Compared with vehicle, no significant changes were observed in the percentage of CD3^+^ T cells, CD4^+^ T cells, CD8^+^ T cells, or CD19^+^ B cells between experimental groups.

#### Bone marrow

No significant changes in absolute cell counts (Fig. 3 D) or frequency of immune subsets (data not shown) were detected in the bone marrow between experimental groups.

Collectively, absolute counts of total CD3^+^ T cells as well as CD4^−^/CD8^−^ DNTs, a biomarker of ALPS, in the spleen, lymph nodes, and blood were significantly lower in mice receiving 80 mg/kg leniolisib. In the spleen, mice administered 80 mg/kg also had significantly lower absolute counts of CD4^+^ T cells and CD19^+^ B cells.

### Mice treated with leniolisib had lower absolute white blood cell counts, lymphocytes, and monocytes

At the end of the study, terminal blood samples were collected to determine the effect of leniolisib on blood cell counts.

**Figure 4.**
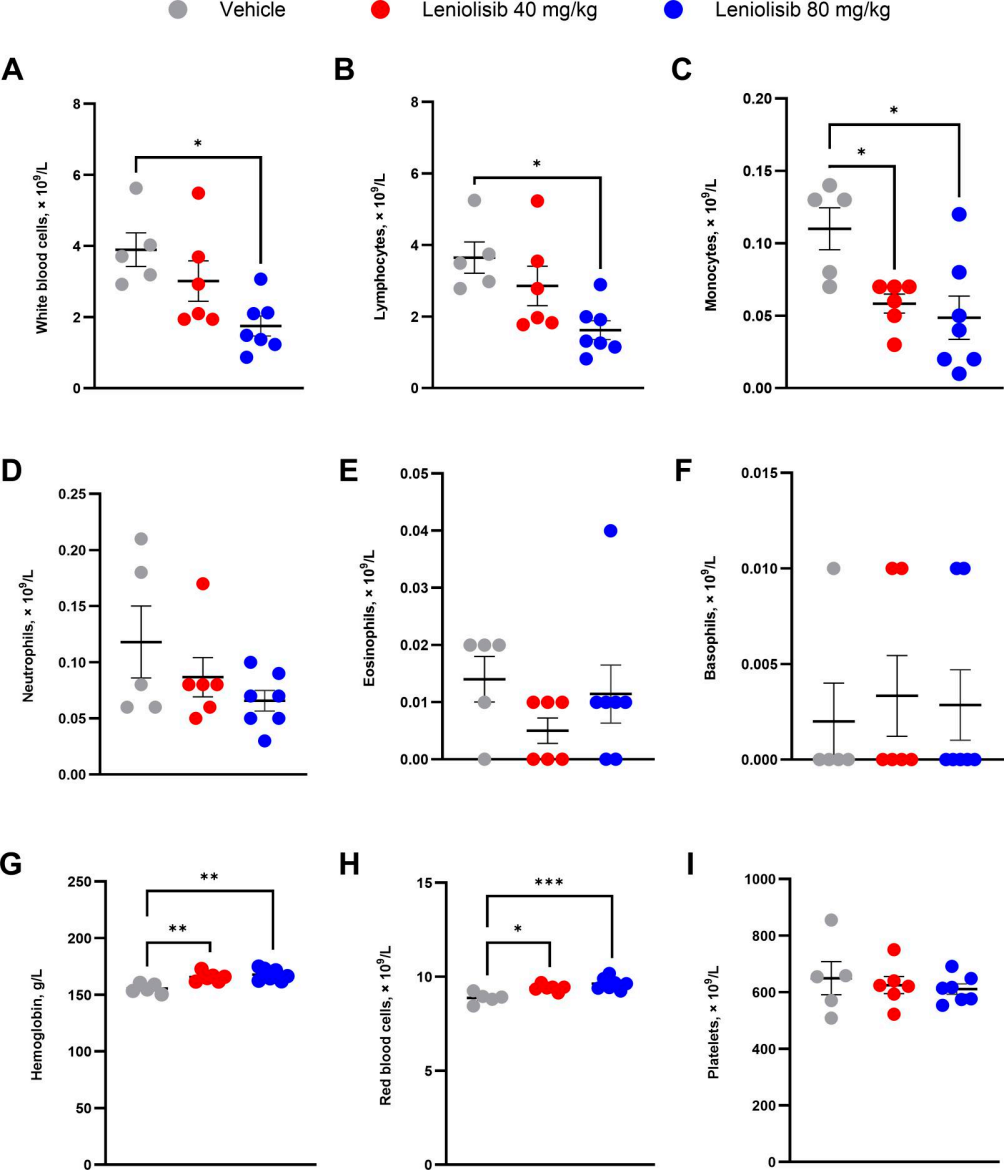
**CBCs and hematologic readouts of terminal blood samples at the end of the study. (A–I)** Individual and mean absolute white blood cell (A), lymphocyte (B), monocyte (C), neutrophil (D), eosinophil (E), and basophil (F) counts. Individual and mean hemoglobin (G), RBCs (H), and platelets (I). *n* values for vehicle, 40 mg/kg leniolisib, and 80 mg/kg leniolisib are 5, 6, and 7, respectively. Error bars are ± SEM. Data were analyzed using a one-way ANOVA with Tukey correction. *P < 0.05; **P < 0.01; ***P < 0.001.

Mice treated with 80 mg/kg leniolisib had significantly lower absolute counts of white blood cells, lymphocytes, and monocytes compared with mice administered vehicle (Fig. 4, A–C). Specifically, mean absolute counts of white blood cells in mice administered 80 mg/kg leniolisib were 1.75 × 10^9^/L vs. 3.90 × 10^9^/L in mice treated with vehicle (P = 0.0106), lymphocytes were 1.62 × 10^9^/L vs. 3.65 × 10^9^/L (P = 0.0110), and monocytes were 0.05 × 10^9^/L vs. 0.11 × 10^9^/L (P = 0.0116). Monocytes were also significantly lower in the 40 mg/kg group (0.06 × 10^9^/L; P = 0.0397). No significant changes were noted in neutrophils, eosinophils, or basophils (Fig. 4, D–F) between experimental groups.

### Leniolisib increased several hematologic parameters

Hemoglobin and hematocrit levels, red blood cell (RBCs) counts, and red cell distribution width (RDW) were significantly higher in mice receiving leniolisib compared with mice administered vehicle, while mean platelet volume was significantly lower. Specifically, hemoglobin levels were 165.6 g/L in the 40 mg/kg leniolisib group (P = 0.0071) and 167.8 g/L in the 80 mg/kg group (P = 0.0011) vs. 155.5 g/L in mice receiving vehicle (Fig. 4 G). Hematocrit levels were 0.50 L/L (P = 0.0100) in mice treated with 40 mg/kg leniolisib and 0.49 L/L in mice treated with 80 mg/kg leniolisib (P = 0.0180) vs. 0.46 L/L in mice receiving vehicle. Absolute counts of RBCs were significantly higher in both leniolisib groups; RBCs were 9.41 × 10^9^/L (P = 0.0137) in mice administered 40 mg/kg leniolisib and 9.63 × 10^9^/L in those administered 80 mg/kg leniolisib (P = 0.0007) vs. 8.87 × 10^9^/L in mice treated with vehicle (Fig. 4 H). In mice treated with 80 mg/kg leniolisib, RDW was significantly higher compared with mice receiving vehicle (18.23% vs. 16.66%; P = 0.0007; data not shown). Mean platelet volume was significantly lower in mice treated with 40 mg/kg leniolisib compared with those treated with vehicle (5.41 fL vs. 5.94 fL; P = 0.0050; data not shown), but no changes were observed in the number of platelets (Fig. 4 I). Finally, no significant changes were observed in mean cell levels of hemoglobin, concentration of hemoglobin, or RBC volume compared with vehicle (data not shown).

### Leniolisib reduced total urine protein

**Figure 5.**
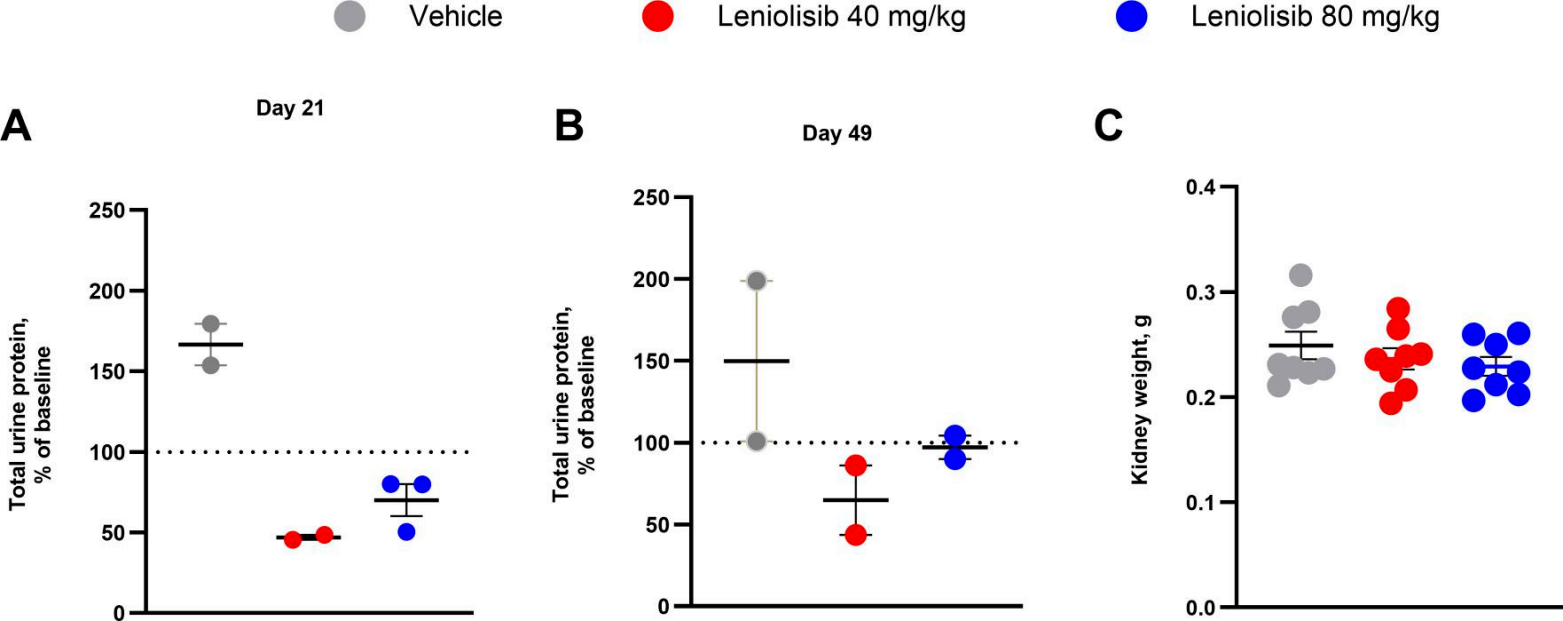
**Total urine protein in pooled urine samples over time and kidney weight at the end of the study. (A–C)** Percent change in total urine protein from baseline values was assessed at day 21 (A) and day 49 (B) of the study. Individual and mean kidney weight (C) at the end of the study. As a minimum of 150 μl of urine was required for total protein analysis, urine samples were pooled in A and B. Pooled samples were extracted from one to four mice per experimental group, and protein analysis was completed in two to three replicates. One kidney (right side) from each mouse was used to determine weight (C). Error bars are ± SEM. Data in C were analyzed using a one-way ANOVA with Tukey correction (P < 0.05).

In addition to outcomes related to ALPS, total urine protein was assessed at baseline and at 21 and 49 days of treatment as a proxy to determine the impact of leniolisib on renal function. MRL/lpr^−/−^ mice often experience progressive renal failure and have been utilized as a model of lupus nephritis (11, 23, 24, 25). Leniolisib treatment groups showed a stabilization or reduction in total urine protein, while the vehicle group experienced an expected increase due to development of the disease (Fig. 5, A and B) (23). Specifically, the percent change in total protein from baseline decreased in mice receiving 40 and 80 mg/kg leniolisib but increased in mice receiving vehicle. At day 21, 40 mg/kg leniolisib decreased total urine protein to 47.0% of the baseline value, and 80 mg/kg decreased it to 70.2% of the baseline value vs. an increase to 166.7% of the baseline value with vehicle. At day 49, total urine protein decreased to 64.9% of the baseline value, and 80 mg/kg decreased it to 97.1% of the baseline value compared with an increase to 150% of the baseline value with vehicle. No significant differences between experimental groups were observed in kidney weight (Fig. 5 C) nor when kidney weight was normalized to body weight.

## Discussion

Overall, leniolisib induced a dose-related reduction in canonical clinical features of ALPS-FAS in the murine model, including lymphadenopathy, splenomegaly, and elevated CD4^−^/CD8^−^ DNTs in blood, spleen, and lymph nodes, and thus reduced the overall disease burden.

Aberrant DNTs of patients with ALPS-FAS have been reported to exhibit increased activity of the PI3K/mTOR-dependent pathway (5). Therefore, we hypothesized that inhibition of PI3Kδ with leniolisib would improve lymphoproliferation via a reduction in elevated DNTs and other accumulated lymphocyte populations. In this study, the weights of the spleen and lymph nodes along with absolute counts of CD4^−^/CD8^−^ DNTs and CD3^+^ T cells were significantly lower in mice treated with leniolisib when compared with vehicle. In terminal blood samples, absolute counts of CD3^+^ T cells were significantly lower with leniolisib than with vehicle. Compared with vehicle, absolute counts of white blood cells, lymphocytes, and monocytes were significantly lower in mice treated with leniolisib, while RBCs, RDW, and hemoglobin and hematocrit levels were higher. Elevated urine protein levels, which are commonly reported in this mouse model and serve as an indicator of autoimmune glomerulonephritis, were absent in mice receiving leniolisib (23). Importantly, no overt toxicities were reported in mice treated with up to 80 mg/kg/day of leniolisib for 7 wk via oral gavage. Overall, we demonstrated that leniolisib limited disease burden in a murine model of ALPS.

The results from the current preclinical work have significant implications within the clinical domain. Nearly all patients with ALPS-FAS develop lymphadenopathy and splenomegaly and are at an increased risk for developing lymphoma (3). Lymphoproliferation in these patients has been attributed to the accumulation of pathognomonic CD4^−^/CD8^−^ DNTs (3, 26). Seminal studies illustrated that splenectomy can effectively reduce lymphoproliferation in MRL/lpr^−/−^ mice; however, post-splenectomy sepsis is a major cause of morbidity and mortality among patients with ALPS (3, 27). Therefore, we sought to investigate an alternative immunomodulatory approach that could address lymphoproliferation. Oral administration of leniolisib decreased total counts of lymphocytes and DNTs as well as spleen weight in MRL/lpr^−/−^ mice, further suggesting that a reduction in DNTs may be associated with a proportional decrease in lymphoproliferation in ALPS, but future studies are needed to gain a comprehensive understanding of the underlying mechanism (4, 5, 28, 29, 30).

Another prominent manifestation in patients with ALPS is recurrent multilineage cytopenias, which are reported in ∼70% of patients and often require multiple agents for treatment (3). Although the mouse model used in the current study lacks a specific cytopenia phenotype, an increase in hemoglobin and hematocrit levels, absolute counts of RBCs, and RDW support the notion that leniolisib may improve cytopenias for patients with ALPS. Moreover, treatment with the selective PI3Kδ inhibitor parsaclisib improved hemoglobin levels in adults with autoimmune hemolytic anemia, further supporting PI3Kδ inhibition as a potential therapeutic approach for autoimmune cytopenias, which are common and can be refractory in patients with ALPS (4, 31). Given that increased PI3K signaling in lymphocytes has been reported in patients with various autoimmune conditions, we speculate that PI3Kδ inhibitors may improve autoimmune cytopenias by reducing elevated levels of autoantibodies (32). Historically, sirolimus significantly reduced autoantibodies in MRL/lpr^−/−^ mice, further supporting PI3K/mTOR inhibition to improve autoimmunity (30). However, further studies are needed to confirm this.

While nephritis has only been reported in a limited subset of patients with ALPS, it is a known phenotype in MRL/lpr^−/−^ mice (23, 33, 34). As these mice often experience glomerulonephritis, they are commonly used as a preclinical mode of lupus nephritis (35, 36, 37). Consistent with findings from the current preclinical study, leniolisib improved proteinuria in a patient with APDS who experienced refractory lupus nephritis (38). These results support the notion that leniolisib may improve renal manifestations associated with ALPS, such as nephritis, but further studies are needed.

Currently, the treatment landscape for ALPS can be challenging for clinicians to navigate, with many options addressing only specific manifestations or inducing adverse events. For example, most patients with ALPS require treatment at a very young age for refractory autoimmune cytopenias (3). First-line treatment for ALPS often includes corticosteroids, which may lead to serious adverse events, such as hypertension, bone fractures, and avascular necrosis, with long-term use (39, 40). The most common immunomodulatory drugs used for the treatment of ALPS are mycophenolate mofetil (MMF) and sirolimus. In clinical trials, treatment with MMF improved autoimmune cytopenias in patients with ALPS while inducing hypogammaglobulinemia in some; however, MMF had little to no effect on lymphoproliferation or elevated CD4^−^/CD8^−^ DNTs (40, 41). Similarly, sirolimus improved autoimmune cytopenias and organomegaly in patients with ALPS but requires long-term monitoring of serum levels and can induce adverse events such as mucositis, hypertension, and hypertriglyceridemia (6). Therefore, well-tolerated and effective therapeutics are needed to manage manifestations associated with ALPS.

In mice, several treatment approaches targeting autoimmune features have been studied, including immunosuppressive compounds that largely target T cells, antirheumatics, antiparasitics, and surgical procedures such as thymectomy, splenectomy, and allogeneic bone marrow transplantation (13, 15, 17, 18, 42, 43, 44, 45, 46, 47, 48). While these approaches have shed light on the potential mechanisms of autoimmunity in ALPS, some failed to ameliorate manifestations, and many relied on immunosuppressive agents that may not be ideal for long-term use (e.g., valproic acid, pyrimethamine, and MMF) (13, 15, 17). The impact of sirolimus was assessed in both MRL/lpr^−/−^ and CBA-lpr^cg^ mice (30, 49). In the latter, mice administered sirolimus experienced a reduction in lymphoproliferation and CD4^−^/CD8^−^ DNTs (30). In MRL/lpr^−/−^ mice, sirolimus reduced lymphoproliferation and urine protein levels, but its effect on CD4^−^/CD8^−^ DNTs was not reported (49). In our current work, we illustrated leniolisib can not only improve elevated urine protein levels and limit lymphoproliferation in MRL/lpr^−/−^ mice but can also limit the expansion of CD4^−^/CD8^−^ DNTs.

Considering leniolisib does not require strict monitoring of serum levels and was well tolerated in patients ≥12 years of age with APDS, we are optimistic that this selective PI3Kδ inhibitor could prove to be an effective treatment for patients, especially in children with ALPS who experience significant lymphoproliferation (8, 50). Currently, a clinical trial is recruiting patients with either germline or somatic variants in *FAS* (NCT06549114). The trial aims to assess the safety of leniolisib and examine clinical measures such as cytopenias and lymphoproliferation. Additionally, two pediatric trials are currently assessing the safety and efficacy of leniolisib in pediatric patients with APDS between the ages of 1–6 and 4–11 years (NCT05693129 and NCT05438407, respectively). Notably, leniolisib will be administered to patients 1–6 years of age as granules, which is suitable for children who cannot swallow pills.

This study has several limitations. Despite the potential to limit generalizability but consistent with other studies assessing ALPS-FAS, only female MRL/lpr^−/−^ mice were used in the current proof-of-concept study, as disease manifestations are accelerated and more severe compared with males (41, 49, 51). Additionally, several mice across different experimental groups (6 of 24) were excluded from complete blood cell count (CBC) analysis (see Materials and methods), reducing the sample size for this outcome measure. Similarly, urine protein analyses required samples be pooled from mice, reducing the overall sample size per experimental group for this measure. As patients with ALPS often experience defects in apoptosis, the impact of leniolisib on these mechanisms as well as on levels of interleukin 10, soluble Fas ligand, lipids, vitamin B12, and phosphorylated AKT/S6 levels warrants further investigation. Additionally, as MRL/lpr^−/−^ mice experience autoreactivity that is suspected to accelerate the development of lymphoproliferation and autoimmunity, assessment of autoantibodies in mice receiving leniolisib is another potential area of study (52). These and additional future studies may further build upon the evidence presented here, which suggests that leniolisib may be an effective agent for treatment of children and adults with ALPS.

## Materials and methods

### Animals and leniolisib treatment schedule

Experimental support was provided by Sygnature Discovery. This work was approved by the Institutional Animal Welfare and Ethical Review Body and carried out under the United Kingdom Home Office Project License in accordance with the Animals Scientific Procedures Act of 1986 and the European Directive 2010/63/EU. A sample size of 7 animals per group was deemed suitable based on a statistical power of 95% and an alpha value of 0.05. An additional animal was included per group for a total of 8 mice per experimental group to mitigate risk of data loss due to disease-related mortality. All experiments in this in vivo study with 8 mice per treatment group were conducted once. 6-wk-old female MRL/MpJ-Fas^lpr^/J (MRL/lpr^−/−^) mice (strain # 000485; Jackson Laboratories) received a daily dose of leniolisib (40 or 80 mg/kg) or vehicle via oral gavage for 7 wk (all groups, *n* = 8). Vehicle consisted of 0.5% (weight per weight) methylcellulose and 0.5% (weight per weight) Tween 80 (Polysorbate 80) aqueous solution.

### Clinical observations and sample collection

All animals had a full health check once daily that included palpation of lymph nodes to check for lymphadenopathy. Body weight was determined daily for the duration of the study. Urine samples were collected at day −1 (baseline) and at days 21 and 49 post treatment and pooled within experimental groups (a minimum of 150 μl was required for protein analysis). Pooled samples were extracted from one to four mice per experimental group, and urine analysis was completed in two to three replicates.

24 h after the final dose, mice were euthanized, and terminal blood, bone marrow, spleens, lymph nodes, and kidneys were collected. Terminal blood samples were collected from all animals. Part of the sample was used for CBC analysis, while the other was used for flow cytometry. Both femurs and half of the spleen from each mouse were collected and stored in phosphate-buffered saline (lot #20012027; Gibco) on ice prior to analysis. Six lymph nodes (two inguinal, two axillary, and two brachial) from each mouse were weighed, while three from each mouse were combined for flow cytometry analysis. One kidney from each mouse was weighed.

### Flow cytometry analysis

Study samples were processed and stained for flow cytometry analysis immediately after necropsy.

**Figure S3.**
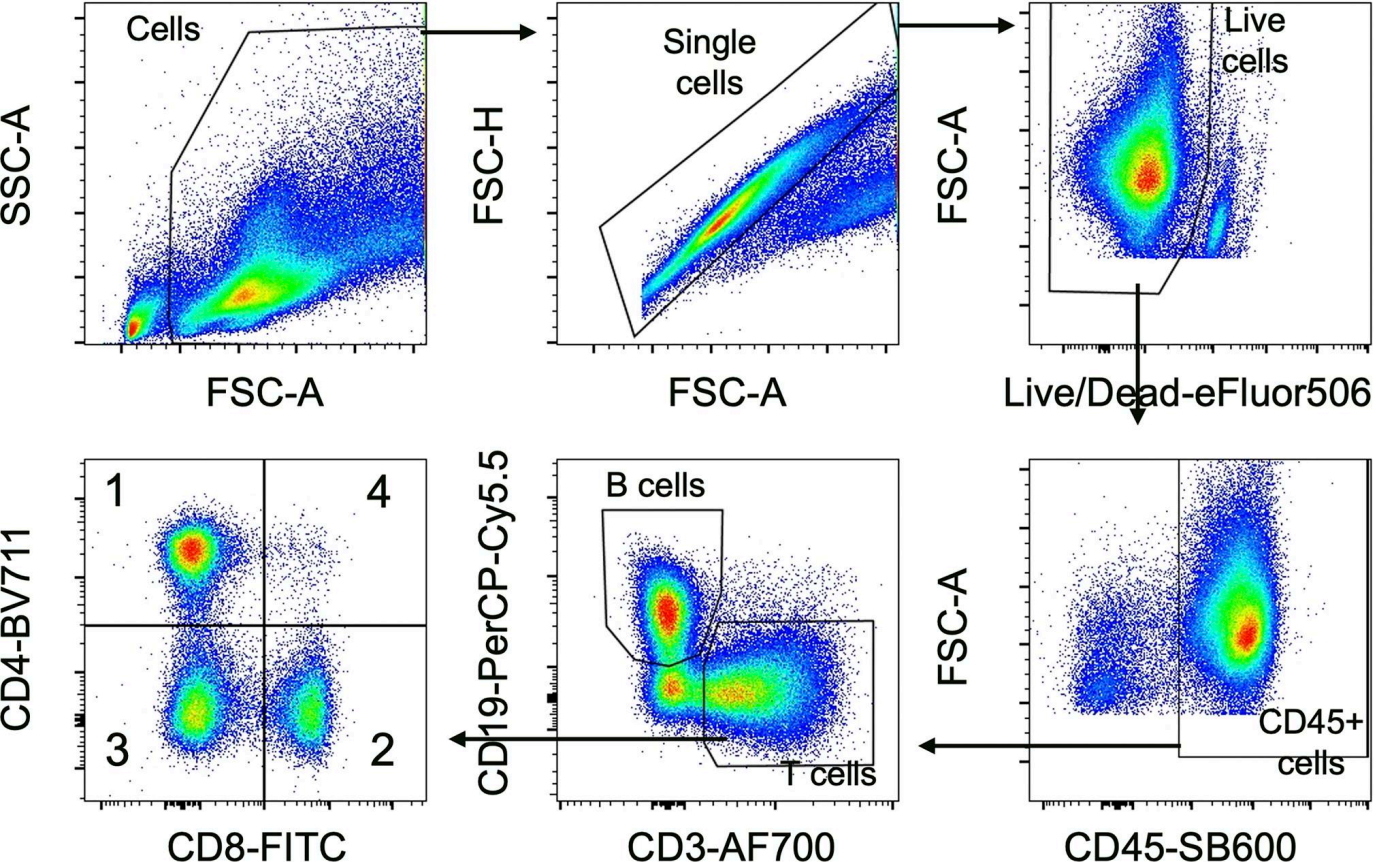
**Representative flow cytometry sample gating for the spleen.** AF, Alexa Fluor; BV, Brillant Violet; FITC, fluorescein isothiocyanate; FSC-A, forward scatter area; FSC-H, forward scatter height; PerCP-Cy5.5, peridinin chlorophyll protein Cy5.5; SB, SuperBright; SSC-A, side scatter area.

Blood, spleen, lymph nodes, and bone marrow cellularity were assessed by isolating single-cell suspensions and counting live cells using live/dead fixable viability dye (diluted 1:1,000, catalog #65-0866-14; eBioscience). Lymphocyte subsets were assessed via flow cytometry of single-cell suspensions using the following antibodies: CD45 (diluted 1:500, clone 30-F11, catalog #63-0451-82; eBioscience), CD4 (diluted 1:500, clone RM4-5, catalog #100557; BioLegend), CD8 (diluted 1:100, clone 53-6.7, catalog #100706; BioLegend), CD19 (diluted 1:1,000, clone 1D3, catalog #152405; BioLegend), and CD3 (diluted 1:40, clone 17A2, catalog #56-0032-82; eBioscience). Markers used to identify lymphocyte subsets were as follows: B cells, CD45^+^CD19^+^; CD4^+^ T cells, CD3^+^CD4^+^CD8^−^; CD8^+^ T cells, CD3^+^CD8^+^CD4^−^; DNTs, CD3^+^CD4^−^CD8^−^. Total CD3^+^ T cells included CD4^+^, CD8^+^, and DNTs. Samples were examined using an Attune NxT flow cytometer (Thermo Fisher Scientific) within 24 h of staining. Representative sample gating for the spleen can be found in Fig. S3. Compensation matrices for each panel were determined using UltraComp eBeads (lot #01-2222-41; Thermo Fisher Scientific). Flow cytometry standard files (.fcs) were analyzed using FlowJo v10.8.1 software.

### Total urine protein analysis

Total urine protein expressed as a percentage of baseline at the midpoint (21 days) and end-of-study (49 days) was determined using the turbidimetric method. Absorbance was measured at 505 nm. Urine samples were analyzed within a week of collection.

### CBC analysis

Terminal blood samples were collected and analyzed within 24 h of collection using the Coulter Principle (53). A DxH 520 Hematology Analyzer (lot #B40602; Beckman Coulter) was used to count the individual cells and provide cell size distribution. Collectively, 6 of 24 mice were excluded (leniolisib, *n* = 3; vehicle, *n* = 3) from CBC analysis due to insufficient blood volume (*n* = 3) or visible clots (*n* = 3).

### Data analysis

All graphs were plotted using GraphPad Prism v10.2.3. Statistical analyses were performed using either a one- or two-way ANOVA with Tukey correction for multiple comparisons. A P value < 0.05 with 95% confidence was considered significant.

### Online supplemental material

Fig. S1 illustrates the frequency of live cells at the end of the study in the spleen, lymph nodes, blood, and bone marrow. Fig. S2 details changes in the frequency of select immune subsets at the end of the study in the spleen, lymph nodes, and blood. Fig. S3 shows representative flow cytometry sample gating for the spleen.

## Data Availability

The data are available upon reasonable request. Researchers who have a methodologically sound research proposal should send inquiries or requests to info@pharming.com. Data requestors may be required to sign a data access agreement.

## References

[1] Dowdell, K.C., J.E. Niemela, S. Price, J. Davis, R.L. Hornung, J.B. Oliveira, J.M. Puck, E.S. Jaffe, S. Pittaluga, J.I. Cohen, . 2010. Somatic FAS mutations are common in patients with genetically undefined autoimmune lymphoproliferative syndrome. Blood. 115:5164–5169. 10.1182/blood-2010-01-263145 20360470 PMC2892951

[2] Magerus, A., A. Rensing-Ehl, V.K. Rao, D.T. Teachey, F. Rieux-Laucat, and S. Ehl. 2024. Autoimmune lymphoproliferative immunodeficiencies (ALPIDs): A proposed approach to redefining ALPS and other lymphoproliferative immune disorders. J. Allergy Clin. Immunol. 153:67–76. 10.1016/j.jaci.2023.11.004 37977527 PMC10841637

[3] Price, S., P.A. Shaw, A. Seitz, G. Joshi, J. Davis, J.E. Niemela, K. Perkins, R.L. Hornung, L. Folio, P.S. Rosenberg, . 2014. Natural history of autoimmune lymphoproliferative syndrome associated with FAS gene mutations. Blood. 123:1989–1999. 10.1182/blood-2013-10-535393 24398331 PMC3968385

[4] Bride, K., and D. Teachey. 2017. Autoimmune lymphoproliferative syndrome: More than a FAScinating disease. F1000Res. 6:1928. 10.12688/f1000research.11545.1 29123652 PMC5668920

[5] Völkl, S., A. Rensing-Ehl, A. Allgäuer, E. Schreiner, M.R. Lorenz, J. Rohr, C. Klemann, I. Fuchs, V. Schuster, A.O. von Bueren, . 2016. Hyperactive mTOR pathway promotes lymphoproliferation and abnormal differentiation in autoimmune lymphoproliferative syndrome. Blood. 128:227–238. 10.1182/blood-2015-11-685024 27099149

[6] Bride, K.L., T. Vincent, K. Smith-Whitley, M.P. Lambert, J.J. Bleesing, A.E. Seif, C.S. Manno, J. Casper, S.A. Grupp, and D.T. Teachey. 2016. Sirolimus is effective in relapsed/refractory autoimmune cytopenias: Results of a prospective multi-institutional trial. Blood. 127:17–28. 10.1182/blood-2015-07-657981 26504182 PMC4705607

[7] Joenja [package insert] . 2023. Pharming Technologies BV, Leiden, Netherlands.

[8] Rao, V.K., S. Webster, A. Sediva, A. Plebani, C. Schuetz, A. Shcherbina, N. Conlon, T. Coulter, V.A. Dalm, A. Trizzino, . 2023. A randomized, placebo-controlled phase 3 trial of the PI3Kδ inhibitor leniolisib for activated PI3Kδ syndrome. Blood. 141:971–983. 10.1182/blood.2022018546 36399712 PMC10163280

[9] Nagata, S. 1998. Human autoimmune lymphoproliferative syndrome, a defect in the apoptosis-inducing Fas receptor: A lesson from the mouse model. J. Hum. Genet. 43:2–8. 10.1007/s100380050029 9609991

[10] Watanabe, T., Y. Sakai, S. Miyawaki, A. Shimizu, O. Koiwai, and K. Ohno. 1991. A molecular genetic linkage map of mouse chromosome 19, including the lpr, Ly-44, and Tdt genes. Biochem. Genet. 29:325–335. 10.1007/BF00554140 1684099

[11] Watson, M.L., J.K. Rao, G.S. Gilkeson, P. Ruiz, E.M. Eicher, D.S. Pisetsky, A. Matsuzawa, J.M. Rochelle, and M.F. Seldin. 1992. Genetic analysis of MRL-lpr mice: Relationship of the Fas apoptosis gene to disease manifestations and renal disease-modifying loci. J. Exp. Med. 176:1645–1656. 10.1084/jem.176.6.1645 1460423 PMC2119463

[12] Cohen, P.L., and R.A. Eisenberg. 1991. Lpr and gld: Single gene models of systemic autoimmunity and lymphoproliferative disease. Annu. Rev. Immunol. 9:243–269. 10.1146/annurev.iy.09.040191.001331 1910678

[13] Dowdell, K.C., L. Pesnicak, V. Hoffmann, K. Steadman, A.T. Remaley, J.I. Cohen, S.E. Straus, and V.K. Rao. 2009. Valproic acid (VPA), a histone deacetylase (HDAC) inhibitor, diminishes lymphoproliferation in the Fas -deficient MRL/lpr(−/−) murine model of autoimmune lymphoproliferative syndrome (ALPS). Exp. Hematol. 37:487–494. 10.1016/j.exphem.2008.12.002 19217201 PMC2693256

[14] Goidl, E.A., R.A. Good, G.W. Siskind, M.E. Weksler, and G. Fernandes. 1986. Studies of immune responses in mice prone to autoimmune disorders. II. Decreased down-regulation by auto-anti-idiotype antibody in autoimmune-prone mice. Cell. Immunol. 101:281–289. 10.1016/0008-8749(86)90141-3 3489534

[15] Jonsson, C.A., M. Erlandsson, L. Svensson, J. Mölne, and H. Carlsten. 1999. Mycophenolate mofetil ameliorates perivascular T lymphocyte inflammation and reduces the double-negative T cell population in SLE-prone MRLlpr/lpr mice. Cell. Immunol. 197:136–144. 10.1006/cimm.1999.1570 10607431

[16] Nagata, N., R. Yasumizu, Y. Ohnishi, T. Nakagawa, M. Inaba, and S. Ikehara. 1990. Auto-MHC class II-reactive T cell line obtained from MRL/+mice suffering from “lpr-GVHD”. I. Characterization of surface phenotypes, specificities and functions in vitro. Immunobiology. 181:367–378. 10.1016/S0171-2985(11)80505-X 2099906

[17] Rao, V.K., K.C. Dowdell, J.K. Dale, F. Dugan, L. Pesnicak, L.L. Bi, V. Hoffmann, S. Penzak, N.A. Avila, T.A. Fleisher, . 2007. Pyrimethamine treatment does not ameliorate lymphoproliferation or autoimmune disease in MRL/lpr^−/−^ mice or in patients with autoimmune lymphoproliferative syndrome. Am. J. Hematol. 82:1049–1055. 10.1002/ajh.21007 17674358

[18] Shimomatsu, T., N. Kanazawa, N. Mikita, Y. Nakatani, H.J. Li, Y. Inaba, T. Ikeda, T. Kondo, and F. Furukawa. 2016. The effect of hydroxychloroquine on lupus erythematosus-like skin lesions in MRL/lpr mice. Mod. Rheumatol. 26:744–748. 10.3109/14397595.2016.1140711 26873035

[19] Volk, H.D., and R.V. Baehr. 1984. The basis of autoimmunity in MRL-lpr/lpr mice and man. Immunol. Today. 5:257–258. 10.1016/0167-5699(84)90133-6 25290322

[20] Watanabe-Fukunaga, R., C.I. Brannan, N.G. Copeland, N.A. Jenkins, and S. Nagata. 1992. Lymphoproliferation disorder in mice explained by defects in Fas antigen that mediates apoptosis. Nature. 356:314–317. 10.1038/356314a0 1372394

[21] Baggott, J.E., S.L. Morgan, L.E. Freeberg, B.B. Hudson, W.H. Vaughn, M. Gopal Nair, C.L. Krumdieck, W.J. Koopman, R.E. Gay, and S. Gay. 1992. Long-term treatment of the MRL/lpr mouse with methotrexate and 10-deazaaminopterin. Agents Actions. 35:104–111. 10.1007/BF01990959 1509970

[22] Furukawa, F., H. Kanauchi, H. Wakita, Y. Tokura, T. Tachibana, Y. Horiguchi, S. Imamura, S. Ozaki, and M. Takigawa. 1996. Spontaneous autoimmune skin lesions of MRL/n mice: Autoimmune disease-prone genetic background in relation to Fas-defect MRL/1pr mice. J. Invest. Dermatol. 107:95–100. 10.1111/1523-1747.ep12298305 8752846

[23] Kelley, V.E., and J.B. Roths. 1985. Interaction of mutant lpr gene with background strain influences renal disease. Clin. Immunol. Immunopathol. 37:220–229. 10.1016/0090-1229(85)90153-9 4042431

[24] Mihara, M., and Y. Ohsugi. 1992. Autoimmune kidney disease in MRL/lpr mice inhibited by OK-432; II. Effect of indomethacin. J. Pharmacobiodyn. 15:255–259. 10.1248/bpb1978.15.255 1527701

[25] Zhao, Y., Z. Zheng, X. Jin, S. Liang, C. Zhang, M. Zhang, Y. Lang, P. Li, and Z. Liu. 2025. Aurora kinase B inhibitor AZD1152: Repurposing for treatment of lupus nephritis driven by the results of clinical trials. eBioMedicine. 112:105553. 10.1016/j.ebiom.2024.105553 39799765 PMC11773216

[26] Kakkanaiah, V.N., M. Nagarkatti, J.A. Bluestone, and P.S. Nagarkatti. 1991. CD4−CD8− thymocytes from MRL-lpr/lpr mice exhibit abnormal proportions of alpha beta- and gamma delta-TCR+ cells and demonstrate defective responsiveness when activated through the TCR. Cell. Immunol. 137:269–282. 10.1016/0008-8749(91)90078-p 1832583

[27] Smathers, P.A., T.J. Santoro, T.M. Chused, J.P. Reeves, and A.D. Steinberg. 1984. Studies of lymphoproliferation in MRL-lpr/lpr mice. J. Immunol. 133:1955–1961. 6332142

[28] Li, X., D. Guo, I.X. Zou, L. Zhao, N. Yang, and Y. Liu. 2025. CD3^+^CD4^−^CD8^−^ T cells: A new potential therapeutic target in treating autoimmune diseases. Front. Immunol. 16:1683418. 10.3389/fimmu.2025.1683418 41080560 PMC12510931

[29] Teachey, D.T., R. Greiner, A. Seif, E. Attiyeh, J. Bleesing, J. Choi, C. Manno, E. Rappaport, D. Schwabe, C. Sheen, . 2009. Treatment with sirolimus results in complete responses in patients with autoimmune lymphoproliferative syndrome. Br. J. Haematol. 145:101–106. 10.1111/j.1365-2141.2009.07595.x 19208097 PMC2819393

[30] Teachey, D.T., D.A. Obzut, K. Axsom, J.K. Choi, K.C. Goldsmith, J. Hall, J. Hulitt, C.S. Manno, J.M. Maris, N. Rhodin, . 2006. Rapamycin improves lymphoproliferative disease in murine autoimmune lymphoproliferative syndrome (ALPS). Blood. 108:1965–1971. 10.1182/blood-2006-01-010124 16757690 PMC1895548

[31] Barcellini, W., F. Pane, A. Patriarca, I. Murakhovskaya, L. Terriou, M.T. DeSancho, W.T. Hanna, L. Leopold, E. Rappold, K. Szeto, . 2024. Parsaclisib for the treatment of primary autoimmune hemolytic anemia: Results from a phase 2, open-label study. Am. J. Hematol. 99:2313–2320. 10.1002/ajh.27493 39435908

[32] Bier, J., and E.K. Deenick. 2022. The role of dysregulated PI3Kdelta signaling in human autoimmunity. Immunol. Rev. 307:134–144. 10.1111/imr.13067 35092042

[33] Glerup, M., T. Herlin, S. Rittig, K. Grønbæk, M. Hokland, and H. Hasle. 2013. Tubulointerstitial nephritis in a patient with probable autoimmune lymphoproliferative syndrome. J. Pediatr. Hematol. Oncol. 35:e187–e189. 10.1097/MPH.0b013e31828ac9fe 23588339

[34] Wieczorkiewicz-Plaza, A., K. Bernat-Sitarz, B. Bienias, K. Kalicka, and P. Sikora. 2017. Glomerulonephritis in course of autoimmune lymphoproliferative syndrome – a case report. Ann. Acad. Med. Siles. 71:104–108. 10.18794/aams/69417

[35] Keil, A., S.R. Hall, M. Körner, M. Herrmann, R.A. Schmid, and S. Frese. 2016. Suppression of lupus nephritis and skin lesions in MRL/lpr mice by administration of the topoisomerase I inhibitor irinotecan. Arthritis Res. Ther. 18:243. 10.1186/s13075-016-1144-5 27770825 PMC5075215

[36] Renner, K., F.J. Hermann, K. Schmidbauer, Y. Talke, M. Rodriguez Gomez, G. Schiechl, J. Schlossmann, H. Brühl, H.-J. Anders, and M. Mack. 2015. IL-3 contributes to development of lupus nephritis in MRL/lpr mice. Kidney Int. 88:1088–1098. 10.1038/ki.2015.196 26131743

[37] Wang, H., M. Lu, S. Zhai, K. Wu, L. Peng, J. Yang, and Y. Xia. 2019. ALW peptide ameliorates lupus nephritis in MRL/lpr mice. Arthritis Res. Ther. 21:261. 10.1186/s13075-019-2038-0 31791413 PMC6889545

[38] Lim, J.P., M. Ahmadian, H. Du, C. Rickert, T. Ghosh, F. Xing, S.A. Johnson, J.P. Weinman, N. Willard, C. Lin, . 2025. Precision medicine in pediatric autoimmunity: Leniolisib treatment of childhood-onset lupus nephritis due to activated phosphoinositide 3-kinase delta syndrome. Arthritis Rheumatol. 77:1596–1604. 10.1002/art.43254 40386960

[39] Rice, J.B., A.G. White, L.M. Scarpati, G. Wan, and W.W. Nelson. 2017. Long-term systemic corticosteroid exposure: A systematic literature review. Clin. Ther. 39:2216–2229. 10.1016/j.clinthera.2017.09.011 29055500

[40] Teachey, D.T., and M.P. Lambert. 2013. Diagnosis and management of autoimmune cytopenias in childhood. Pediatr. Clin. North Am. 60:1489–1511. 10.1016/j.pcl.2013.08.009 24237984 PMC5384653

[41] Rao, V.K., F. Dugan, J.K. Dale, J. Davis, J. Tretler, J.K. Hurley, T. Fleisher, J. Puck, and S.E. Straus. 2005. Use of mycophenolate mofetil for chronic, refractory immune cytopenias in children with autoimmune lymphoproliferative syndrome. Br. J. Haematol. 129:534–538. 10.1111/j.1365-2141.2005.05496.x 15877736

[42] Asensi, V., K. Kimeno, I. Kawamura, M. Sakumoto, and K. Nomoto. 1989. Treatment of autoimmune MRL/lpr mice with anti-B220 monoclonal antibody reduces the level of anti-DNA antibodies and lymphadenopathies. Immunology. 68:204–208. 2478453 PMC1385418

[43] Gonzalez, N.M., D. Zou, Z. Zeng, F.X. Feng, X. Zhang, C. Sannes, A. Gu, Y. Zu, and W. Chen. 2025. Transient anti-TCRβ mAb treatment induces CD4 ^+^ T cell exhaustion and prolongs survival in a mouse model of systemic lupus erythematosus. Immunology. 174:239–246. 10.1111/imm.13881 39648274

[44] Ikehara, S., R. Yasumizu, M. Inaba, S. Izui, K. Hayakawa, K. Sekita, J. Toki, K. Sugiura, H. Iwai, and T. Nakamura. 1989. Long-term observations of autoimmune-prone mice treated for autoimmune disease by allogeneic bone marrow transplantation. Proc. Natl. Acad. Sci. USA. 86:3306–3310. 10.1073/pnas.86.9.3306 2654943 PMC287120

[45] Jabs, D.A., C.L. Burek, Q. Hu, R.C. Kuppers, B. Lee, and R.A. Prendergast. 1992. Anti-CD4 monoclonal antibody therapy suppresses autoimmune disease in MRL/Mp-lpr/lpr mice. Cell. Immunol. 141:496–507. 10.1016/0008-8749(92)90166-m 1576659

[46] Thomas, T.J., U.B. Gunnia, and T. Thomas. 1992. Reversal of the abnormal development of T cell subpopulations in the thymus of autoimmune MRL-lpr/lpr mice by a polyamine biosynthesis inhibitor. Autoimmunity. 13:275–283. 10.3109/08916939209112336 1472637

[47] Wofsy, D., J.A. Ledbetter, J.R. Roubinian, W.E. Seaman, and N. Talal. 1982. Thymic influences on autoimmunity in MRL-lpr mice. Scand. J. Immunol. 16:51–58. 10.1111/j.1365-3083.1982.tb00698.x 6981841

[48] Yamamoto, K., A. Mori, T. Nakahama, M. Ito, H. Okudaira, and T. Miyamoto. 1990. Experimental treatment of autoimmune MRL-lpr/lpr mice with immunosuppressive compound FK506. Immunology. 69:222–227. 1689694 PMC1385593

[49] Warner, L.M., L.M. Adams, and S.N. Sehgal. 1994. Rapamycin prolongs survival and arrests pathophysiologic changes in murine systemic lupus erythematosus. Arthritis Rheum. 37:289–297. 10.1002/art.1780370219 8129783

[50] Rao, V.K., E. Kulm, A. Sediva, A. Plebani, C. Schuetz, A. Shcherbina, V.A. Dalm, A. Trizzino, Y. Zharankova, S. Webster, . 2024. Interim analysis: Open-label extension study of leniolisib for patients with APDS. J. Allergy Clin. Immunol. 153:265–274.e9. 10.1016/j.jaci.2023.09.032 37797893 PMC10841669

[51] Jeltsch-David, H., and S. Muller. 2014. Neuropsychiatric systemic lupus erythematosus and cognitive dysfunction: The MRL-lpr mouse strain as a model. Autoimmun. Rev. 13:963–973. 10.1016/j.autrev.2014.08.015 25183233

[52] Weston, K.M., E.T. Yeh, and M.S. Sy. 1987. Autoreactivity accelerates the development of autoimmunity and lymphoproliferation in MRL/Mp-lpr/lpr mice. J. Immunol. 139:734–742. 3110283

[53] Graham, M.D. 2022. The coulter Principle: A history. Cytometry. A. 101:8–11. 10.1002/cyto.a.24505 34611994

